# Histidine tracts in human transcription factors: insight into metal ion coordination ability

**DOI:** 10.1007/s00775-017-1512-x

**Published:** 2017-12-07

**Authors:** Aleksandra Hecel, Joanna Wątły, Magdalena Rowińska-Żyrek, Jolanta Świątek-Kozłowska, Henryk Kozłowski

**Affiliations:** 1 Faculty of Chemistry, University of Wroclaw, F. Joliot-Curie 14, 50-383 Wrocław, Poland; 2 Public Higher Medical Professional School in Opole, Katowicka 68, 45-060 Opole, Poland; 3 Wroclaw Research Centre EIT+, Stabłowicka 147, 54-066 Wrocław, Poland

**Keywords:** Binding affinity, Ligand binding, Thermodynamics, Homeostasis, Mass spectrometry, Peptide

## Abstract

Consecutive histidine repeats are chosen both by nature and by molecular biologists due to their high affinity towards metal ions. Screening of the human genome showed that transcription factors are extremely rich in His tracts. In this work, we examine two of such His-rich regions from forkhead box and MAFA proteins—MB3 (contains 18 His) and MB6 (with 21 His residues), focusing on the affinity and binding modes of Cu^2+^ and Zn^2+^ towards the two His-rich regions. In the case of Zn^2+^ species, the availability of imidazole nitrogen donors enhances metal complex stability. Interestingly, an opposite tendency is observed for Cu^2+^ complexes at above physiological pH, in which amide nitrogens participate in binding.

**Electronic supplementary material:**

The online version of this article (10.1007/s00775-017-1512-x) contains supplementary material, which is available to authorized users.

## Introduction

Histidine-rich (His-rich) motifs are present in many peptide domains and consist of multiple His residues, which can efficiently bind metal ions [1–4]. They have been found in a variety of proteins such as metal transporters [5, 6], prion proteins [7–11], bacterial nickel chaperones [12–18], snake venom proteins [19, 20], antimicrobial peptides [21, 22], histidine-rich glycoproteins (HRG) [23, 24] and many others of biological significance. Repeats of a different number of histidines are common also in commercially used immobilized metal affinity chromatography (IMAC) and they are known as His-tags [25].They are specific type of His-rich sequences, usually containing from six to nine subsequent histydyl residues and used in molecular biology for purification of recombinant proteins [26].

The widespread occurrence of histidine repeats in nature is very exciting from the evolutional, biological, chemical and medical point of view. Understanding their interactions with metal ions is not only chemically intriguing, but is also the fundamental step towards using them in potential medicinal applications [27–29]. Single amino acid repeats are extremely important in eukaryotic proteins [30]. This homopolymeric tracts are known to play important structural or even functional roles. Indeed, there is an over representation of single amino acid repeat (SAR)-containing proteins among transcription factors, kinases and proteins required for development [31–34]. Among homopolymeric tracts, sequences with histidine-tag (His-tag) motif (special sequences containing a huge number of neighboring His residues) are relatively rare [34]. These repeats may fulfill different roles, affecting protein conformation and enzymatic activity—His-tags are found in Zn-finger domains involved in interactions between nucleic acids and proteins [35]; flexible His tracts have been found in transcriptional regulators [36, 37] and as nuclear speckle-targeting signals [38].

86 proteins in the human genome contain stretches of five or more consecutive histidine residues; most of these proteins have functions related with RNA synthesis; their mechanism of action is not clear [39]. Our recent studies on different ‘poly-His’ region interactions with Cu^2+^ and Zn^2+^ [1, 2, 4, 40–42] show that they form thermodynamically stable complexes with so-called polymorphic binding states, with the metal “moving back and forth” along such regions [1, 2]. It is worth to notice that in many cases, metal ion binding induced the formation of an α-helical structure [2].

Understanding the relationship between metal ion binding, structure and function is one of the most important pillars of bioinorganic chemistry. Sequences with consecutive His repeats have been chosen both by nature and by molecular biologists as metal chelators due to their high affinity towards metal ions. Understanding Cu^2+^ and Zn^2+^ coordination to our studied sequences will be an important input to the bioinorganic chemistry of the studied metals that allows a better understanding of the proper design of His-tags. To the best of our knowledge, thermodynamics of metal complexes with protein sequences containing 18 and 22 His residues have not yet been studied.

This work explains the interactions of Cu^2+^ and Zn^2+^ with two multihistidine peptide fragments from transcription factors: MB3 (Ac-HHASHGHHNSHHPQHHHHHHHHHHH-NH_2_), the 33–57 region of FOXG1B (forkhead box) protein, which plays an important role in the regional subdivision of the developing brain [43] and MB6 (Ac-HHHGAHHAAHHHHAAHHHHHHHHHSHGGAGHGGGAGHH-NH_2_), a 184–219 region of the MAFA protein, which specifically activates insulin expression [44, 45]; phosphorylation is required for its oncogenic activity—it can function either as an oncogene or as a tumor suppressor, depending on the cell context [46]. A combination of mass spectrometric, potentiometric and spectroscopic studies show the coordination abilities of these ligands towards Cu^2+^ and Zn^2+^ ions.

## Experimental

### Materials

The N- and C-terminally protected MB3 (Ac-HHASHGHHNSHHPQHHHHHHHHHHH-NH_2_) and MB6 (Ac-HHHGAHHAAHHHHAAHHHHHHHHHSHGGAGHGGGAGHH-NH_2_) fragments were purchased from KareBayBiochem (USA) (certified purity 98%) and used as received. Their purity was checked potentiometrically. Cu(ClO_4_)_2_ and Zn(ClO_4_)_2_ were extra pure products (Sigma-Aldrich); concentration of their stock solutions was determined by ICP–MS. The carbonate-free stock solution of 0.1 mol dm^−3^ NaOH was potentiometrically standardized with potassium hydrogen phthalate (both Sigma-Aldrich). All samples were prepared with freshly doubly distilled water. The ionic strength (*I*) was adjusted to 0.1 M by addition of NaClO_4_ (Sigma-Aldrich).

### Mass spectrometric measurements

High-resolution mass spectra were obtained on Bruker MicrOTOF-Q spectrometers (Bruker Daltonik, Bremen, Germany) equipped with an Apollo II electrospray ionization source with an ion funnel. Spectrometer was used for measurements on Cu^2+^ and Zn^2+^ complexes (with both ligands) in the range of positive values. The instrumental parameters were as follows: scan range *m/z* 250–2000; dry gas nitrogen; temperature 200 °C; ion source voltage 4500 V; collision energy 10 eV. The Cu^2+^ and Zn^2+^ complexes [(metal:ligand stoichiometry of 1:1.2 and 1:2, respectively), [ligand]_tot_ = 2 × 10^−4^ M] were prepared in a 1:1 MeOH/H_2_O mixture at pH 6 (by adding an appropriate amount of NaOH). The samples were infused at a flow rate of 3 μL/min. Before each experiment, the instrument was calibrated externally with the Tunemix mixture. Data were processed by application of the Bruker Compass DataAnalysis 4.0. program.

### Potentiometric measurements

Stability constants for proton and Cu^2+^ and Zn^2+^ complexes were calculated basing on two titration curves carried out over the pH range 2–11 at 298 K in a total volume of 3 cm^3^. The potentiometric titrations were performed using a Dosimat 665 Metrohm titrator connected to a Metrohm 691 pH-meter and a Metrohm LL Unitrode glass electrode. The glass cell was equipped with a magnetic stirring system, a microburet delivery tube and an inlet–outlet tube for argon. The pH-metric titrations were performed in 30% DMSO solution of HClO_4_ at 0.1 M NaClO_4_ ionic strength (both ligands are insoluble in pure water solution). Solutions were titrated with 0.1 M carbonate-free NaOH. Electrodes were calibrated daily for hydrogen ion concentration by titrating HClO_4_ with KOH in the same experimental conditions as above. Purities and the exact concentrations of ligand solutions were determined by the Gran method [47]. The ligand concentration was 0.5 mM. Metal ions concentration were 0.42 mM Cu^2+^ and 0.25 mM Zn^2+^, respectively. The metal-to-ligand ratio was 1:1.2 for Cu^2+^ complexes and 1:2 for Zn^2+^ complexes. HYPERQUAD2006 and SUPERQUAD programs were used for the stability constant calculations [48]. Standard deviations were computed by HYPERQUAD 2006 and refer to random errors only. The constants for hydrolytic Cu^2+^ and Zn^2+^ species were used [49, 50]. The speciation and competition diagrams were computed with the HySS program [51].

### Spectroscopic studies

Circular dichroism (CD) spectroscopy experiments were performed on a spectropolarimeter Jasco-J-750 at 298 K in a 10 mm quartz cell. The spectral range was 250–800 nm. Samples were prepared in 4.0 mM HClO_4_ (30% DMSO solutions containing 0.1 M NaClO_4_ ionic strength). Ligand concentration was 1 mM and Cu^2+^ to ligand molar ratio was 1:1.2. The direct CD measurements (*Θ*) were converted to mean residue molar ellipticity (Δ*ε*) using Jasco Spectra Manager.

The absorption spectra in the UV–Vis region were recorded at 298 K on a Varian Cary 300 Bio spectrophotometer in 10 mm path length quartz cell. The spectral range was 200–800 nm. The samples were prepared in 4.0 mM HClO_4_ (30% DMSO solutions containing 0.1 M NaClO_4_ ionic strength). Ligand concentration was 1 mM and Cu^2+^ to ligand molar ratio was 1:1.2.

Electron paramagnetic resonance (EPR) spectra were recorded in liquid nitrogen on a Bruker ELEXSYS E500 CW-EPR spectrometer at X-band frequency (9.5 GHz) and equipped with an ER 036TM NMR Teslameter and an E41 FC frequency counter. The ligands were prepared in 30% DMSO solution of HClO_4_ at *I* = 0.1 M (NaClO_4_). The concentration of Cu^2+^ was 1 mM and the *M*:*L* molar ratio was 1:1.2. In the EPR experiment, a natural mixture of ^63^Cu and ^65^Cu isotopes was used, both of them with nuclear spin *I* = 3/2. Ethylene glycol (30%) was used as a cryoprotectant for EPR measurements. The EPR parameters were analyzed by computer simulation of the experimental spectra using WIN-EPR SIMFONIA software, version 1.2 (Bruker). The pH was adjusted with appropriate amounts of HCl and NaOH solutions. A mixture of copper isotopes was used, which never give separate signals in case of nitrogen and/or oxygen coordinating donors, but lead to broadening of the signals; the line around perpendicular component of g tensor is also broadened due to poorly resolved copper hyperfine splitting. This resonance transition reveals the best resolution of ^14^N hyperfine splitting, what is well known from the [52–58]. Since both DMSO and water solvents are very improper for the observation of nitrogen hyperfine splitting (leading to strong absorption of microwaves and giving very weak EPR spectra), frozen solutions were used.

## Results and discussion

Structural and thermodynamic properties of Cu^2+^– and Zn^2+^–MB3 and MB6 complexes were studied by mass spectrometric, potentiometric calculations and a variety of spectroscopic techniques: UV–Vis, CD and EPR spectroscopy. Potentiometric titrations were the basis for the determination of precise stability constants and pH-dependent species distribution diagrams and combined spectroscopic techniques results allowed to determine the copper and zinc binding modes and the coordination geometries of these species formed in solution.

### Protonation constants of the MB3 (Ac-HHASHGHHNSHHPQHHHHHHHHHHH-NH_2_) and MB6 (Ac-HHHGAHHAAHHHHAAHHHHHHHHHSHGGAGHGGGAGHH-NH_2_) ligands

Each of the peptide MB3 and MB6 was protected in the N-terminus by acetylation and in the C-terminus by amidation. MB3 consists of eighteen possible sites of protonations—all of them are assigned to the eighteen histidine residues (Table S1). The MB6 peptide consists of 22 sites (Table S1) involved in acid–base equilibria, which correspond to imidazole nitrogen atoms of histidine residues. Because of the enormous number of histidine residues in presented sequences, not all protonation constants could be precisely determined. During potentiometric measurements, His residues deprotonate in the pH range of 4–8 and it is not possible to observe (or rather to precisely calculate) the p*K*a of each from—they probably deprotonate in pairs. The log*β* values obtained from potentiometric titration analysis are typical values of histidine residues in poly-His systems [1, 4]. The distribution diagrams of investigated ligands are presented on Figures S1 and S2.

### Metal binding stoichiometry of the Cu^2+^/Zn^2+^–MB3 system

Electrospray ionization mass spectrometry (ESI–MS) confirmed the purity of the studied MB3 ligand (Ac-HHASHGHHNSHHPQHHHHHHHHHHH-NH_2_) and showed the metal binding stoichiometry at pH 6, indicating that equimolar species were present under the studied conditions (e.g. *m/z* values at 808.58 and 647.67 correspond to [CuLH_4_]^4+^ and [ZnLH_5_]^5+^ complex species, respectively) (Figure S1). *m/z* values at 793.10, 634.68 and 529.07 correspond to [LH_4_]^4+^, [LH_5_]^5+^, [LH_6_]^6+^ ligand species, respectively (Figure S1 A).

### Cu^2+^–MB3 system

**Fig. 1:**
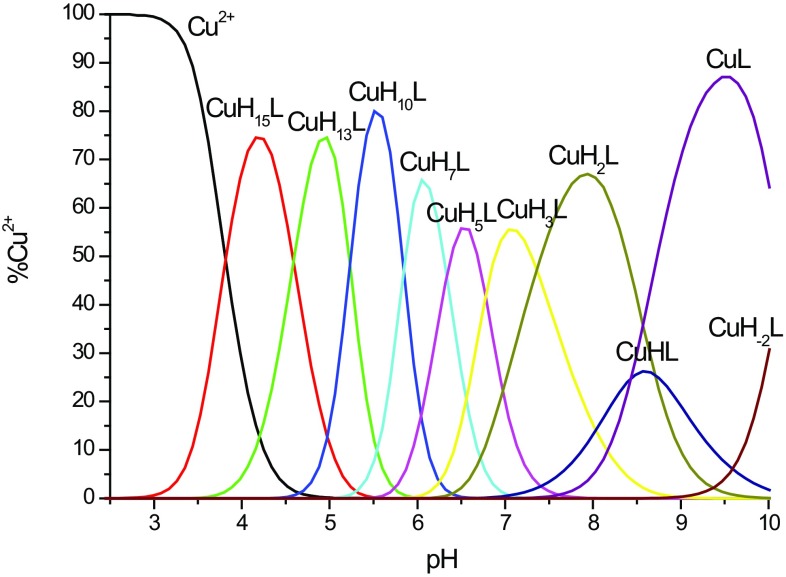
Species distribution diagram for Cu^2+^–MB3 complexes at 1:1.2 Cu^2+^/peptide ratio; *T* = 298 K; *c*_peptide_ = 0.5 mM (for clarity, the charges on the speciation plots were omitted)

Potentiometric titrations of Cu^2+^–MB3 complexes were carried out to evaluate the corresponding complex formation constants and the distribution diagram (Table 1, Fig. 1).

**Table 1:** Potentiometric and spectroscopic data for Cu^2+^–MB3 complexes

Complex species	log*β*	UV–Vis		CD		EPR		Proposed donors
		*λ* (nm)	*ε* (M^−1^ cm^−1^)	*λ* (nm)	Δ*ε* (M^−1^ cm^−1^)	$A_{\parallel }$ [G]	$g_{\parallel }$	
Cu^2+^–Ac-HHASHGHHNSHHPQHHHHHHHHHHH-NH_2_								
[CuH_15_L]^15+^	101.75 (2)	620	50.56	527	0.80	181.1	2.245	3N_im_
[CuH_13_L]^13+^	92.57 (3)	568	68.00	534	1.51	190.1	2.244	3N_im_, 1N^−^
		315	222.93	313	− 0.71			
[CuH_10_L]^10+^	76.87 (3)	568	70.42	534	1.64	192.1	2.244	3N_im_, 1N^−^
		316	227.28	316	− 0.78			
[CuH_7_L]^7+^	59.33 (3)	571	74.48	535	1.68	192.8	2.241	3N_im_, 1N^−^
		317	240.49	317	− 0.79			
[CuH_5_L]^5+^	46.66 (4)							
[CuH_3_L]^3+^	33.11 (5)							
[CuH_2_L]^2+^	25.75 (6)							
[CuHL]^+^	17.04 (13)							
[CuL]	8.61 (5)							
[CuH_−2_L]^2−^	−11.73 (6)	551	111.68	637	1.35	184.1	2.216	2N_im_, 2N^−^
				507	− 0.57			
				406	0.20			
				351	− 0.19			

Cu^2+^ to ligand ratio of 1:1.2. [Cu^2+^] = 0.83 mM

In the studied pH range (2–10), the most accurate fit of titration curves for the Cu^2+^–MB3 complexes indicates the presence of nine equimolar species: [CuH_15_L]^15+^, [CuH_13_L]^13+^, [CuH_10_L]^10+^, [CuH_7_L]^7+^, [CuH_5_L]^5+^, [CuH_3_L]^3+^, [CuH_2_L]^2+^, [CuHL]^+^, [CuL], [CuH_−2_L]^2−^ (Fig. 1). The first species of this complex occurs at pH 4.2 and last of them achieves a maximum concentrations at pH around 10. Careful study of obtained experimental potentiometric and spectroscopic studies allowed a detailed thermodynamic and structural characterization of the complex, showing the number and type of coordinated atoms from the peptide (Table 1).

The first complex detected at low pH is [CuH_15_L]^15+^, with a maximum concentration at pH 4.2 (Fig. 1). It is most probable that in this complex three imidazole residues are coordinated to the Cu^2+^ ion [59]. The coordination of Cu^2+^ to three imidazole nitrogens is supported by the d–d band at 620 nm at pH 3.87 (Figure S4). The shift of the d–d band from 620 to 568 nm in pH range 4–6 suggests the coordination of a fourth nitrogen atom resulting in the {3N_im_, 1N^−^} binding mode for the [CuH_13_L]^13+^, [CuH_10_L]^10+^ and [CuH_7_L]^7+^ species. The coordination of an amide nitrogen is provided by the appearance of intense d–d bands in CD spectra at 530–650 nm range (Figure S5). The coordination of amide nitrogen to Cu^2+^ is also supported by the increase intensity of the characteristic band at 313 nm [50, 60–62]. EPR parameters at pH 4–6 support the suggested four nitrogen coordination modes for the copper complexes, but also suggest the presence of a trace amount of 3N coordinated species, being in equilibria with the 4N complex (Table 1). Unfortunately, due to the precipitation observed from pH around 6.4–9 we were not able to record spectroscopic spectra in this pH range. At pH above 9, the differences observed in the UV–Vis and CD spectra support coordination with further amide nitrogen. The coordination mode for [CuH_−2_L]^2−^ is {2N_im_, 2N^−^} supported by the shift of the d–d band from 570 to 551 nm (Figure S4) and appearance of intense d–d band at around 500 and 640 nm on CD spectra (Figure S5). Comparison of the experimental EPR spectra for Cu^2+^–MB3 systems at pH 6 and 9–10 show the differences in superhyperfine splitting patterns and especially the changes in the values of $A_{\parallel }$, $g_{\parallel }$ and $g^{ \bot }$ parameters, confirming the change of the coordination of three and four nitrogen atoms to the Cu^2+^ ion, respectively [56–58] (Figure S6 and Table 1). The comparison between splitting patterns due to ^14^N hyperfine coupling distinctly reveals the difference between the number of the nitrogen donors in *xy* coordination plane of Cu^2+^ for the complexes formed at pH 6.62 and 9.60 [53–55]. A different number of lines is observed, most probably seven and nine, respectively (Figure S6). Significant differences between *g* and *A* tensor components ($A_{\parallel }$, $g_{\parallel }$ and $g^{ \bot }$) of the species at pH 6.62 and 9.60 are also observed. The values of EPR parameters correspond to three and four nitrogen donors, respectively (according to Peisach and Blumberg’s [52] dependences between $A_{\parallel }$ and $g_{\parallel }$ and the number of nitrogen donors).

### Zn^2+^–MB3 system

**Fig. 2:**
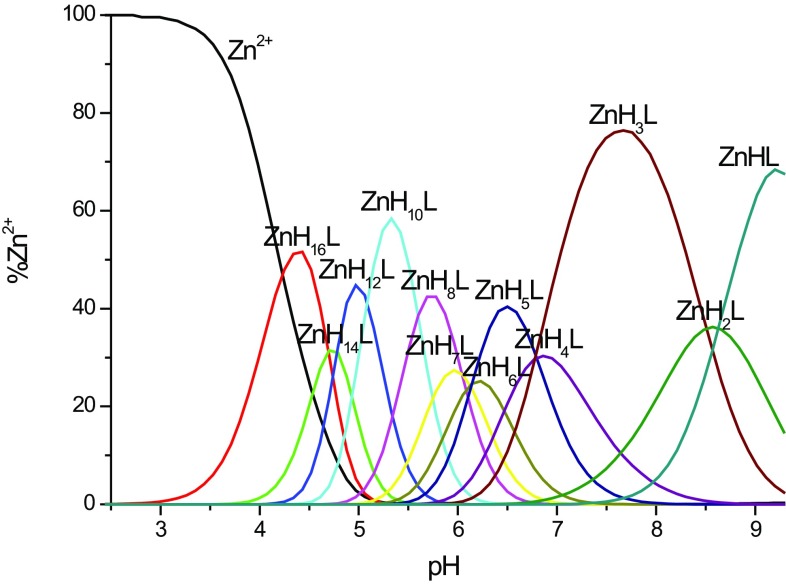
Species distribution diagram for Zn^2+^–MB3 complexes at 1:2 Zn^2+^/peptide ratio; *T* = 298 K; *c*_peptide_ = 0.5 mM. A higher Zn^2+^/peptide ratio triggered precipitation at pH above 8.5. For clarity, charges on the speciation plots were omitted

As in the case of copper complexes, numerous zinc-bound species are observed in the studied pH range. The titration curves for Zn^2+^–MB3 complexes fit best to the formation of the following complexes: [ZnH_16_L]^16+^, [ZnH_14_L]^14+^, [ZnH_12_L]^12+^, [ZnH_10_L]^10+^, [ZnH_8_L]^8+^, [ZnH_7_L]^7+^, [ZnH_6_L]^6+^, [ZnH_5_L]^5+^, [ZnH_4_L]^4+^, [ZnH_3_L]^3+^, [ZnH_2_L]^2+^, [ZnHL]^+^ (Fig. 2). The Zn^2+^ complex formation constants are shown in Table 2. Above pH 9, precipitation is observed.

**Table 2:** Formation constants of Zn^2+^–MB3 complexes at 298 K and *I* = 0.1 M (NaClO_4_)

Complex species	log*β*	p*K*a
[ZnH_16_L]^16+^	104.69 (2)	
[ZnH_14_L]^14+^	95.31 (2)	
[ZnH_12_L]^12+^	85.75 (2)	
[ZnH_10_L]^10+^	75.58 (3)	
[ZnH_8_L]^8+^	64.38 (4)	6.04
[ZnH_7_L]^7+^	58.34 (7)	6.12
[ZnH_6_L]^6+^	52.22 (9)	6.15
[ZnH_5_L]^5+^	46.07 (5)	6.79
[ZnH_4_L]^4+^	39.28(5)	6.78
[ZnH_3_L]^3+^	32.50 (4)	8.51
[ZnH_2_L]^2+^	23.99 (4)	8.63
[ZnHL]^+^	15.36 (6)	

Standard deviations in the last figure are shown in parentheses

In the first complex observed at low pH, [ZnH_16_L]^16+^ with maximum concentration at pH 4.3, most likely two imidazole nitrogens are coordinated to the zinc ion {2N_im_}. In the next species, [ZnH_14_L]^14+^, most probably two other imidazoles bind to the central Zn^2+^ ion, however, due to the lack of spectroscopic data available for d^10^ metal, this statement is suggested only by the decrease of p*K*a for the zinc complex in comparison to the free ligand. The next deprotonations come from other histidine residues which do not participate in binding.

### Metal binding stoichiometry of the Cu^2+^/Zn^2+^–MB6 system

Electrospray ionization mass spectrometry confirmed the purity of the studied MB6 ligand (Ac-HHHGAHHAAHHHHAAHHHHHHHHHSHGGAGHGGGAGHH-NH_2_) and showed the metal binding stoichiometry at pH 6, indicating that only equimolar species were present under the studied conditions (e.g. *m/z* values at 697.46 and 836.55 correspond to [CuLH_6_]^6+^ and [ZnLH_5_]^5+^ complex, respectively), Figure S7. *m/z* values at 1030.21, 687.14, 589.12 and 515.61 correspond to [LH_4_]^4+^, [LH_6_]^6+^, [LH_7_]^7+^ and [LH_8_]^8+^ ligand species, respectively (Figure S7 A).

### Cu^2+^–MB6 system

**Fig. 3:**
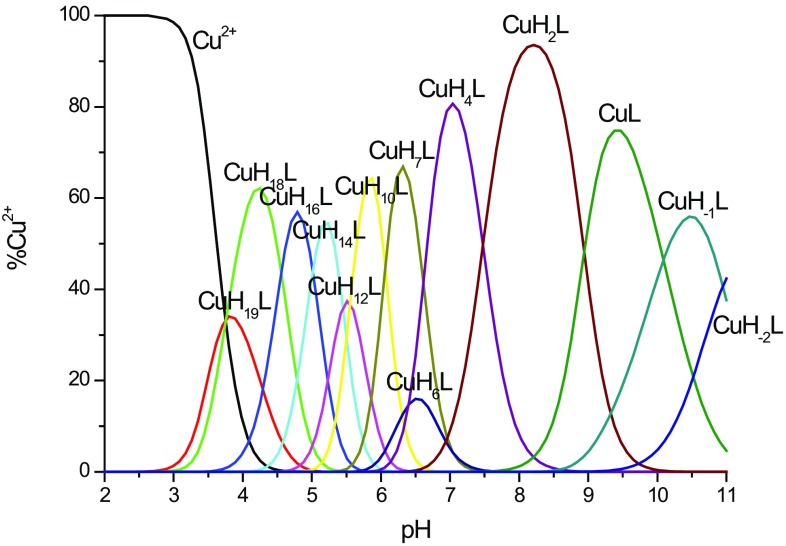
Species distribution diagram for Cu^2+^–MB6 complexes at 1:1.2 Cu^2+^/peptide ratio; *T* = 298 K; *c*_peptide_ = 0.5 mM (for clarity, the charges on the speciation plots were omitted)

Potentiometric measurements revealed thirteen protonated mononuclear Cu^2+^ complexes. Distribution diagrams are shown in Fig. 3 and the corresponding stability constants, together with detailed spectroscopic parameters are reported in Table 3. To investigate the coordination mode of Cu^2+^ with MB6, we used potentiometric titrations and spectroscopic techniques, such as UV–Vis (Figure S8), CD (Figure S9) and EPR (Figure S10). The first Cu^2+^ complex detected with the MB6 ligand is [CuH_19_L]^19+^, with maximum concentration already at pH 3.8. In this complex, two imidazole nitrogens are coordinated to Cu^2+^, which is supported by the d–d band at 687 nm for pH 4 (Figure S8). Next species, [CuH_18_L]^18+^ result from the deprotonation and copper(II) ion binding to the third histidine imidazole—the d–d band shifts from 687 to 618 nm in the {3N_im_} complex. Moreover, EPR parameters are in good agreement with the 3N binding mode (Table 3). The coordination of an amide nitrogen occurs at pH 4.7—the maximum concentration of [CuH_16_L]^16+^ species, as evidenced by the appearance of an intense d–d band in the CD spectra (Figure S9). At pH around 5, a clear shift of the maximum absorption in the direction of shorter wavelengths is observed in the UV–Vis spectra (Figure S8), indicating a {2N_im_, 1N^−^} binding mode (one of the imidazoles is substituted by an amide). For [CuH_14_L]^14+^, [CuH_12_L]^12+^, [CuH_10_L]^10+^, [CuH_7_L]^7+^ and [CuH_6_L]^6+^ species, present at pH range 5.2–6.5, no significant changes are observed in the UV–Vis and CD spectra, suggesting that the {2N_im_, 1N^−^} donor set does not change and the deprotonations correspond to the proton loss of histidines which are not involved in Cu^2+^ binding. Similar to the case of the Cu^2+^–MB3 system, due to the precipitation observed at pH around 6.5–9.3, we were not able to record spectroscopic spectra in this pH range also for Cu^2+^–MB6 complexes. However, because of the presence of ethylene glycol in EPR measurements, which enhanced the complex solubility, we were able to obtain EPR parameters that also confirmed the {2N_im_, 1N^−^} copper binding mode at physiological pH (Table 3, Figure S10). At pH above 9, the differences in the d–d transition energy [a shift of the band to shorter wavelengths and appearance of new d–d bands in the CD spectra (Figures S8 and S9)] strongly support the coordination mode with an additional amide nitrogen—the coordination mode for [CuL], [CuH_−1_L]^−^ and [CuH_−2_L]^2−^ complexes is {2N_im_, 2N^−^}. The 4N coordination is supported also by the EPR parameter $g_{\parallel }$ in the range 2.22–2.205.

**Table 3:** Potentiometric and spectroscopic data for Cu^2+^–MB6 complexes

Complex species	log*β*	UV–Vis		CD		EPR		Proposed donors
		*λ* (nm)	*ε* (M^−1^ cm^−1^)	*λ* (nm)	Δ*ε* (M^−1^ cm^−1^)	$A_{\parallel }$ [G]	$g_{\parallel }$	
Cu^2+^–Ac-HHHGAHHAAHHHHAAHHHHHHHHHSHGGAGHGGGAGHH-NH_2_								
[CuH_19_L]^19+^	123.36 (6)	687	37.80					2N_im_
[CuH_18_L]^18+^	119.60 (2)	618	56.70	533	0.64	186.3	2.24	3N_im_
[CuH_16_L]^16+^	110.48 (3)	571	68.42	536	1.27			2N_im_, 1N^−^
		311	253.15	316	− 0.41			
[CuH_14_L]^14+^	100.46 (2)	569	71.57	533	1.55	186.3	2.24	2N_im_, 1N^−^
		311	255.12	317	− 0.57			
[CuH_12_L]^12+^	89.56 (3)							
[CuH_10_L]^10+^	78.45 (2)	569	76.68	533	1.61			2N_im_, 1N^−^
		311	266.94	316	− 0.66			
[CuH_7_L]^7+^	60.27 (3)					184.2	2.24	2N_im_, 1N^−^
[CuH_6_L]^6+^	53.22 (7)	582	87.10	540	1.18			2N_im_, 1N^−^
		311	313.32					
[CuH_4_L]^4+^	40.42 (3)					171	2.26	2N_im_, 1N^−^
[CuH_2_L]^2+^	25.47 (3)					171	2.26	2N_im_, 1N^−^
[CuL]	7.59 (4)	585	112.20	634	1.34	183	2.22	2N_im_, 2N^−^
				339	− 1.35			
				258	6.90			
[CuH_−1_L]^−^	− 2.50 (3)	555	113.91	632	1.47	184	2.22	2N_im_, 2N^−^
				511	− 0.17			
				342	− 0.91			
				258	7.54			
[CuH_−2_L]^2−^	− 13.45 (8)	534	127.28	638	1.47	190.8	2.205	2N_im_, 2N^−^
				494	− 0.82			
				358	− 0.13			
				305	− 0.23			
				261	8.40			

Cu^2+^ to ligand ratio of 1:1.2. [Cu^2+^] = 0.83 mM

### Zn^2+^–MB6 system

**Fig. 4:**
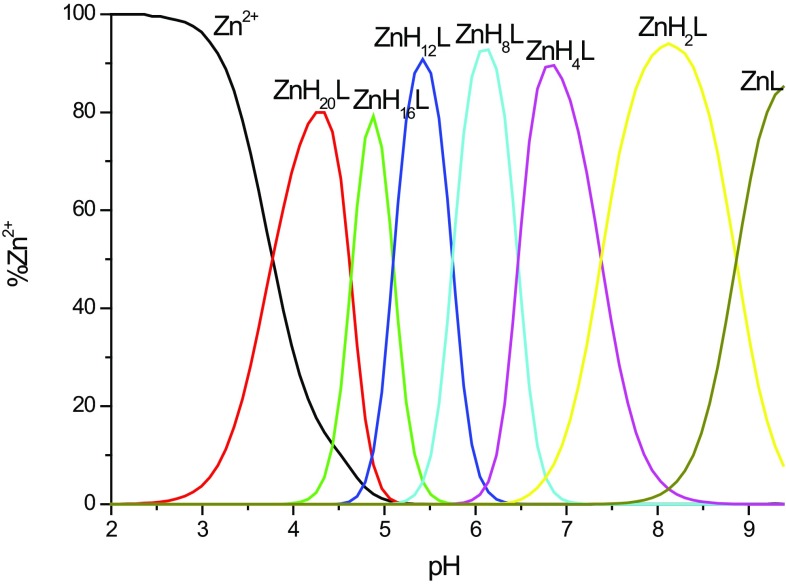
Species distribution diagram for Zn^2+^–MB6 complexes at 1:2 Zn^2+^/peptide ratio; *T* = 298 K; *c*_peptide_ = 0.5 mM. A higher Zn^2+^/peptide ratio triggered precipitation at pH above 8.5. For clarity, charges on the speciation plots were omitted

The MB6 peptide forms seven complex species with Zn^2+^ ions, with the stoichiometry and species distribution shown in Table 4 and Fig. 4. In the Zn^2+^–MB6 system, the first complex species, [ZnH_20_L]^20+^, reaches a maximum concentration at pH 4 (Fig. 4). In this complex, it is expected that two imidazole nitrogen atoms are bound to zinc ions. In the next formed species, [ZnH_16_L]^16+^, most likely another two imidazoles coordinate to the central zinc atom, and two others deprotonate without binding—however, both statements are only a hypothesis, since no stepwise deprotonations are observed and no spectroscopic data are available for the d^10^ metal; it is also probable that three imidazole nitrogens are bound at this point. The next deprotonations most like are due to the loss of protons from unbound His side chains.

**Table 4:** Formation constants of Zn^2+^–MB6 complexes at 298 K and *I* = 0.1 M (NaClO_4_)

Complex species	log*β*
[ZnH_20_L]^20+^	126.83 (10)
[ZnH_16_L]^16+^	108.31 (11)
[ZnH_12_L]^12+^	87.92 (12)
[ZnH_8_L]^8+^	64.94 (12)
[ZnH_4_L]^4+^	39.07 (11)
[ZnH_2_L]^2+^	24.31 (8)
[ZnL]	6.58 (6)

Standard deviations in the last figure are shown in parentheses

## Discussion

**Fig. 5:**
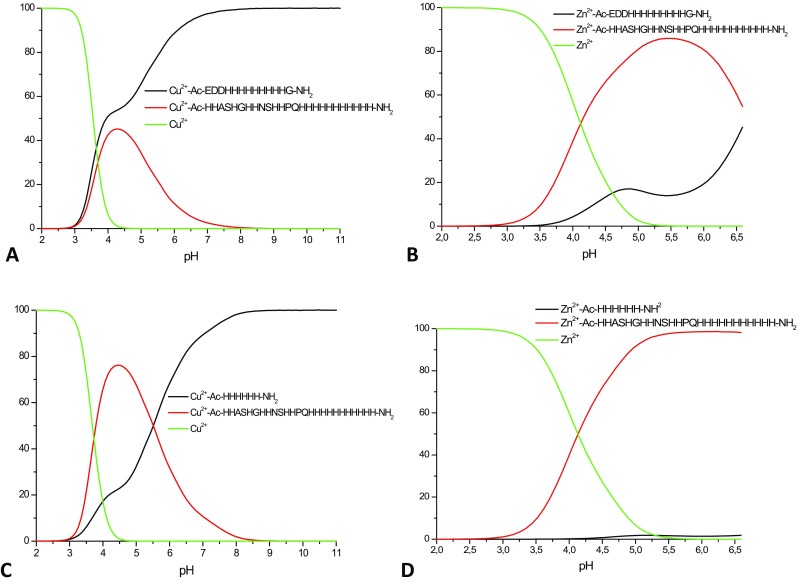
Competition plots for **a** Cu^2+^–Ac-EDDHHHHHHHHHG-NH_2_ and Cu^2+^–Ac-HHASHGHHNSHHPQHHHHHHHHHHH-NH_2_ (MB3); **b** Zn^2+^–Ac-EDDHHHHHHHHHG-NH_2_ and Zn^2+^–Ac-HHASHGHHNSHHPQHHHHHHHHHHH-NH_2_ (MB3); **c** Cu^2+^–Ac-HHHHHH-NH_2_ and Cu^2+^–Ac-HHASHGHHNSHHPQHHHHHHHHHHH-NH_2_ (MB3); **d** Zn^2+^–Ac-HHHHHH-NH_2_ and Zn^2+^–Ac-HHASHGHHNSHHPQHHHHHHHHHHH-NH_2_ (MB3) complexes. Previously calculated stability constants are applied to a theoretical situation, in which equimolar amounts of Cu^2+^/Zn^2+^ and all ligands are present

**Fig. 6:**
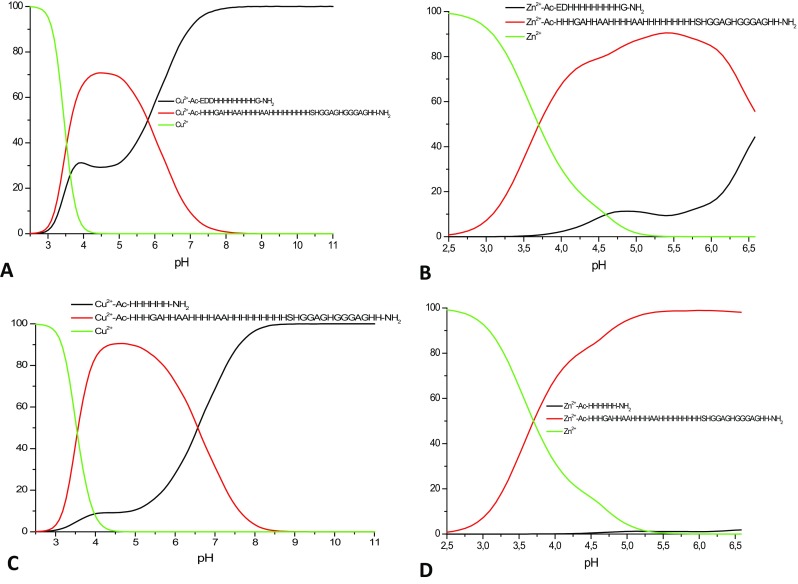
Competition plots for **a** Cu^2+^–Ac-EDDHHHHHHHHHG-NH_2_ and Cu^2+^–Ac-HHHGAHHAAHHHHAAHHHHHHHHHSHGGAGHGGGAGHH-NH_2_ (MB6); **b** Zn^2+^–Ac-EDHHHHHHHHHG-NH_2_ and Zn^2+^–Ac-HHHGAHHAAHHHHAAHHHHHHHHHSHGGAGHGGGAGHH-NH_2_ (MB6); **c** Cu^2+^–Ac-HHHHHH-NH_2_ and Cu^2+^–Ac-HHHGAHHAAHHHHAAHHHHHHHHHSHGGAGHGGGAGHH-NH_2_ (MB6); **d** Zn^2+^–Ac-HHHHHH-NH_2_ and Zn^2+^–Ac-HHHGAHHAAHHHHAAHHHHHHHHHSHGGAGHGGGAGHH-NH_2_ (MB6) complexes. Previously calculated stability constants are applied to a theoretical situation, in which equimolar amounts of Cu^2+^/Zn^2+^ and all ligands are present

What is the impact of the presence of the extraordinarily high number of histidines on the stability of the complexes they form with Cu^2+^ and Zn^2+^ ions? Naturally, the availability of imidazole nitrogen donors is very likely to enhance metal complex stability (even statistically, there is a higher chance to encounter this potential metal binder), but the answer is not as trivial as it may seem. To discuss complex stability, we compared so-called competition plots—they are based on the calculated formation constants and describe a hypothetical situation, in which equimolar amounts of the metal ion and two multi-histidine ligands are present in solution at different pH values (Figs. 5, 6). We compared our MB3 and MB6 ligands with two other His-rich peptides, a typical (His)_6_ tag (Ac-HHHHHH-NH_2_) and a snake venom peptide fragment with nine consecutive histidines (Ac-EDDHHHHHHHHH-NH_2_) [1, 2, 41, 42]. In the case of zinc complexes, the outcome of these comparisons is easier to explain—both our 18-His and 22-His fragments (MB3 and MB6, respectively) bind Zn^2+^ with higher affinity than the His_6_-tag and the 9-His fragment (Figs. 5b, d, 6b, d)—the higher number of potential binding sites enhances complex stability. It is worth to notice that while the His_6_-tag is almost entirely outcompeted by the 18-His and 22-His MB3 and MB6, in the case of the 9-His fragment, the difference is not as striking—this would again confirm the straightforward conclusion—the more histidines, the more stable the zinc complex (Figure S11 A).

The comparison becomes far less trivial in the case of copper(II), which is able to deprotonate and bind to amide nitrogens. For the His_6_-tag, below pH 6, the outcome is easy to interpret—MB3 and MB6 form more stable complexes with Cu^2+^ (Figs. 5c, 6c). For the 9-His fragment, the difference in stability is either not as pronounced (in the case of MB6, Fig. 6a) or comparable (for MB3, Fig. 5a). The really interesting situation starts above pH 6.5—the copper complexes with the shorter fragments become far more stable than the Cu^2+^ ones with 18-His and 22-His MB3 and MB6. How to explain this phenomenon? At this pH, in the case of the two shorter fragments, amide nitrogens start to participate in the binding [1]. Amide binding to Cu^2+^ results in the formation of thermodynamically very stable five and six membered chelate rings, making the complexes more stable than those in which Cu^2+^ is bound to the same number of imidazole nitrogens. Also MB3 has a higher affinity for copper than longer MB6 fragment (Figure S11 B). This is in good agreement with what we already show—less histidines there are in the sequence, the sooner the amides start to participate in the binding and the more stable the complex is. Why do amide nitrogens start bind to Cu^2+^ at lower pH in the case of shorter fragments, with respect to the multihistidine MB3 and MB6 ligands? We can hypothesize that this is due to (1) the presence of so-called polymorphic binding states, where the metal “moves back and forth” along such regions [2]—most likely, the more His residues are present, the more likely the metal is to “move”; (2) stacking interactions within the ligand, which allow amide deprotonation only at higher pH [63].

## Electronic supplementary material

Below is the link to the electronic supplementary material.

Supplementary material 1 (PDF 1227 kb)
