# Supplementary material for: Histidine tracts in human transcription factors: insight into metal ion coordination ability
Source: J Biol Inorg Chem. 2017 Dec 7;23(1):81–90. doi: 10.1007/s00775-017-1512-x (PMC5756558; doi:10.1007/s00775-017-1512-x)
Supplement: Supplementary file 1 — Supplementary material 1 (PDF 1227 kb) [file 775_2017_1512_MOESM1_ESM.pdf]

Histidine tracts in human transcription factors – insight into metal ion coordination ability

Aleksandra Hecel[1*], Joanna Wątły[1], Magdalena Rowińska-Żyrek[1], Jolanta Świątek-Kozłowska[2], Henryk Kozłowski[2,3*]

*[1] Faculty of Chemistry, University of Wroclaw, F. Joliot-Curie 14, 50-383, Wroclaw, Poland**
e-mail: aleksandra.hecel@chem.uni.wroc.pl
[2] Public Higher Medical Professional School in Opole, Katowicka 68, 45-060 Opole, Poland
[3] Wroclaw Research Centre EIT+, Stabłowicka 147, 54-066 Wroclaw, Poland

Table S 1. Protonation constants of A) MB3 (Ac-HHASHGHHNSHHPQHHHHHHHHHHH-NH_2) and B) MB6 (Ac-HHHGAHHAAHHHHAAHHHHHHHHHSHGGAGHGGGAGHH-NH_2) peptides at 298 K and *I*=0.1 M ($NaClO_4$). The standard deviations are reported in parentheses on the last significant figure.

**MB3**	log*β*	**MB6**	log*β*
$[HL]^{+}$	8.99(3)	$[HL]^{+}$	8.05(4)
$[H_2L]^{2+}$	16.43(5)	$[H_2L]^{2+}$	15.82(1)
$[H_3L]^{3+}$	23.66(6)		
$[H_4L]^{4+}$	30.78(6)	$[H_4L]^{4+}$	30.10(2)
$[H_6L]^{6+}$	44.14(6)	$[H_6L]^{6+}$	43.40(2)
$[H_8L]^{8+}$	56.57(6)	$[H_8L]^{8+}$	56.20(2)
$[H_{10}L]^{10+}$	68.51(7)	$[H_{10}L]^{10+}$	68.39(2)
$[H_{12}L]^{12+}$	79.77(7)	$[H_{12}L]^{12+}$	80.19(2)
$[H_{14}L]^{14+}$	90.49(8)	$[H_{14}L]^{14+}$	91.43(2)
$[H_{16}L]^{16+}$	100.53(8)	$[H_{16}L]^{16+}$	102.28(2)
$[H_{18}L]^{18+}$	109.63(9)	$[H_{18}L]^{18+}$	112.52(2)

	$[H_{20}L]^{20+}$	122.23(2)
	$[H_{22}L]^{22+}$	130.91(2)

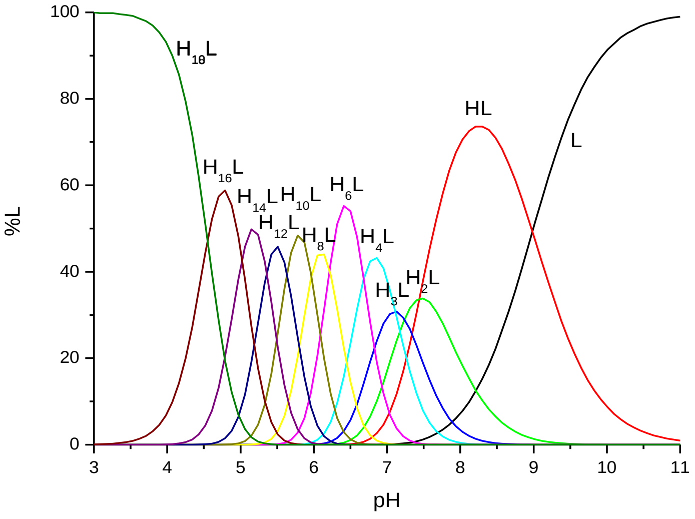

Figure S 1. Species distribution diagram for MB3 peptide; T=298 K; $C_{peptide}$ = 0.5 mM. For clarity, charges on the speciation plots were omitted.

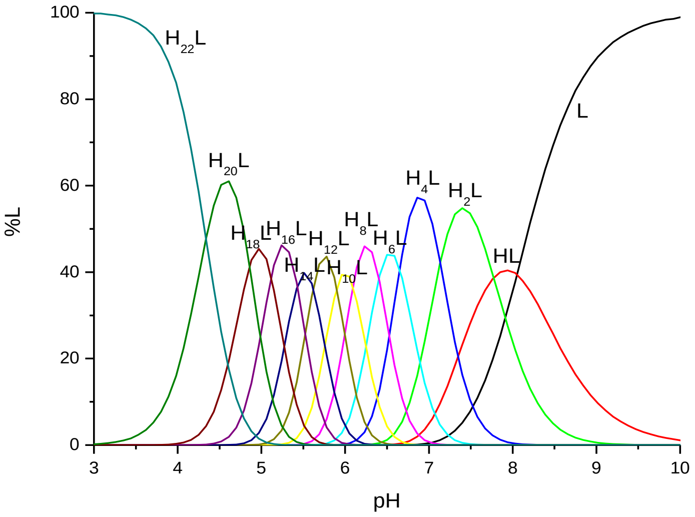

Figure S 2. Species distribution diagram for MB6 peptide; T=298 K; $C_{peptide}$ = 0.5 mM. For clarity, charges on the speciation plots were omitted.

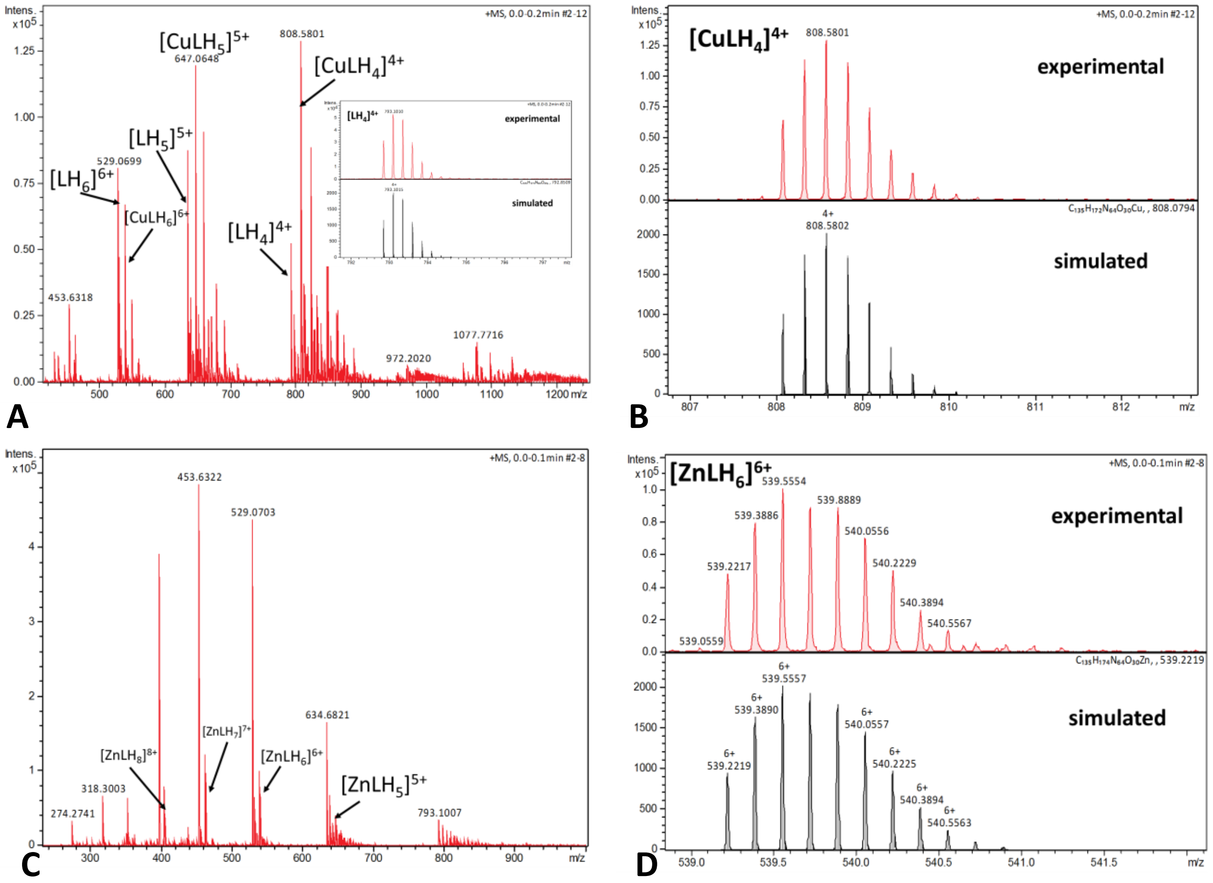

Figure S 3. ESI-MS spectra of the Cu^{2+} – MB3 (A and B) and Zn^{2+} – MB3 (C and D) complexes (ligand to metal stoichiometry of 1:1.1 and 1:2 respectively); $[ligand]_{tot} = 2 \times 10^{-4}$ M; samples prepared in a 1:1 MeOH/H_2O mixture at pH 6; positive ion mode. For chosen complexes a comparison of the experimental and simulated signals was performed.

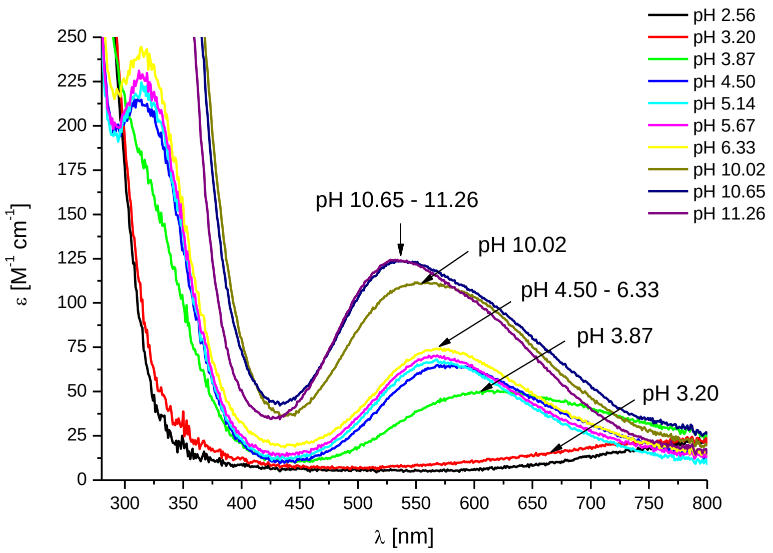

Figure S 4. UV-Vis spectra for Cu^{2+}- MB3 complex in 30% DMSO solution. [Cu^{2+}] = 0.83 mM. Cu^{2+} to ligand ratio of 1:1.2.

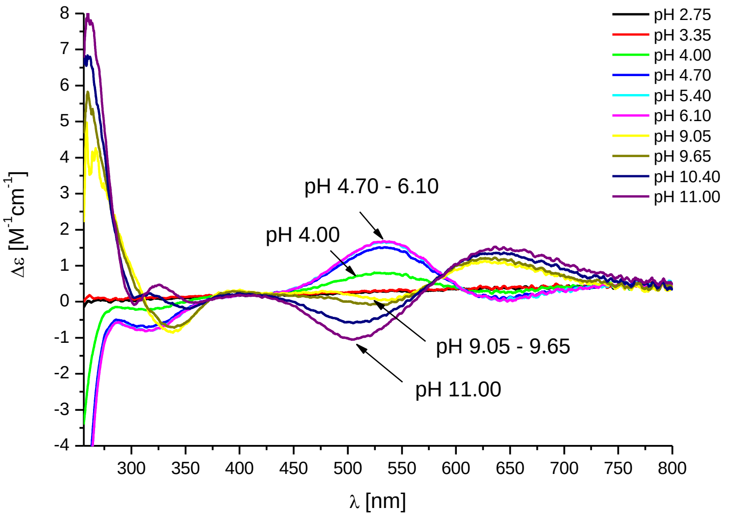

Figure S 5. CD spectra for Cu^{2+}-MB3 complex in 30% DMSO solution. [Cu^{2+}] = 0.83 mM. Cu^{2+} to ligand ratio of 1:1.2. Cuvette path length 10 mm.

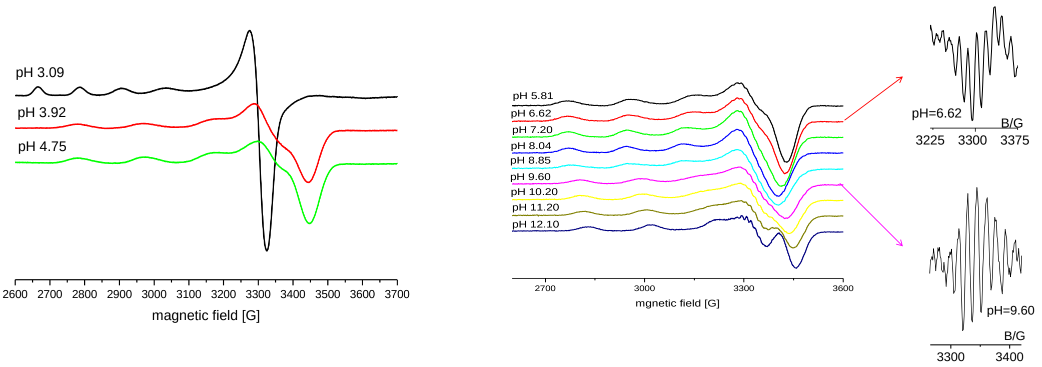

pH	A$_{II}$[G]	g$_{II}$	g$_{\perp}$
3.09	120.0	2.41	2.080
3.92	181.1	2.245	2.065
4.75	190.1	2.244	2.065
5.65	192.1	2.244	2.065

pH	A$_{II}$[G]	g$_{II}$	g$_{\perp}$
5.81	192.8	2.241	2.055
6.62	190.7	2.242	2.055
7.20	179.8	2.255	2.060
8.04	175.4	2.256	2.060
8.85	175.4	2.252	2.060
9.60	181.9	2.225	2.050
10.20	184.1	2.216	2.050
11.20	186.2	2.211	2.045
12.10	190.8	2.20	2.045

Figure S 6. EPR spectra of Cu^{2+}-MB3 system in 30% DMSO solution of ethylene glycol (30%) at different pH values, T = 77 K (X-band – 9.5 GHz). [Cu^{2+}] = 1 mM; molar ratio M:L – 1:1.2. The ^{14}N superhyperfine splitting of the EPR spectrum around g$_{\perp}$ component of g tensor at pH 6.62 and 9.6, at 77 K.; the splitting resolution was enhanced by double differentiation of the spectrum recorded at the same microwave frequency 9.5779 GHz .

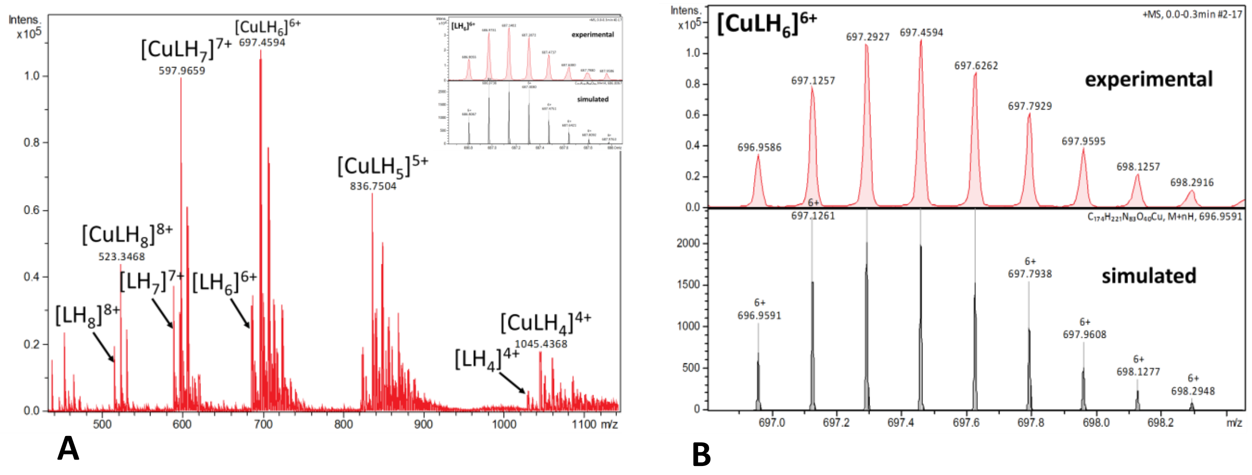

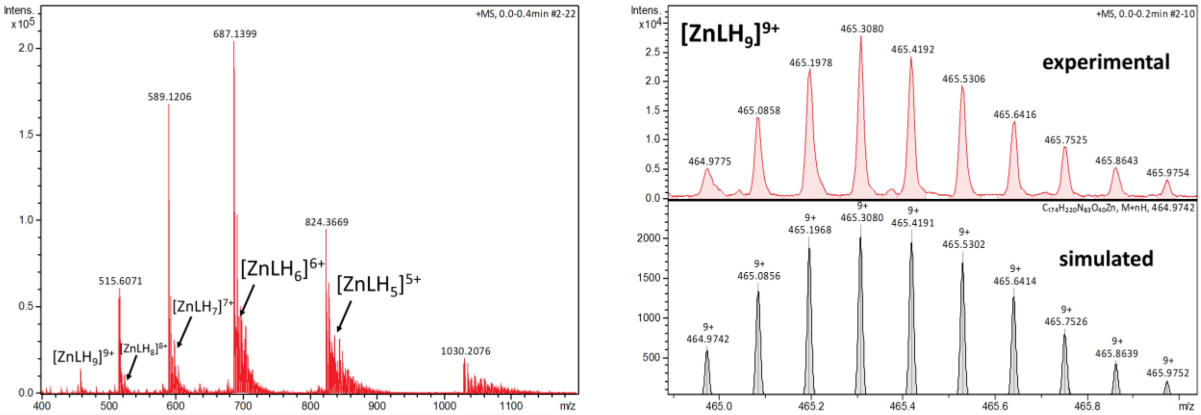

C **D**

Figure S 7. ESI-MS spectra of the Cu^{2+} – MB6 (A and B) and Zn^{2+} – MB6 (C and D)complexes (ligand to metal stoichiometry of 1:1.1 and 1:2 respectively); $[ligand]_{tot}$ = 2 × 10^{-4} M; samples prepared in a 1:1 MeOH/H_2O mixture at pH 6; positive ion mode. For chosen complexes a comparison of the experimental and simulated signals was performed.

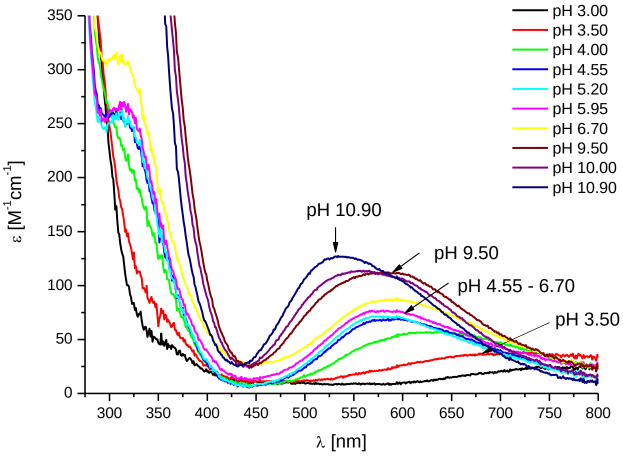

Figure S 8. UV-Vis spectra for Cu^{2+}-MB6 complex in 30% DMSO solution. [Cu^{2+}] = 0.83 mM. Cu^{2+} to ligand ratio of 1:1.2.

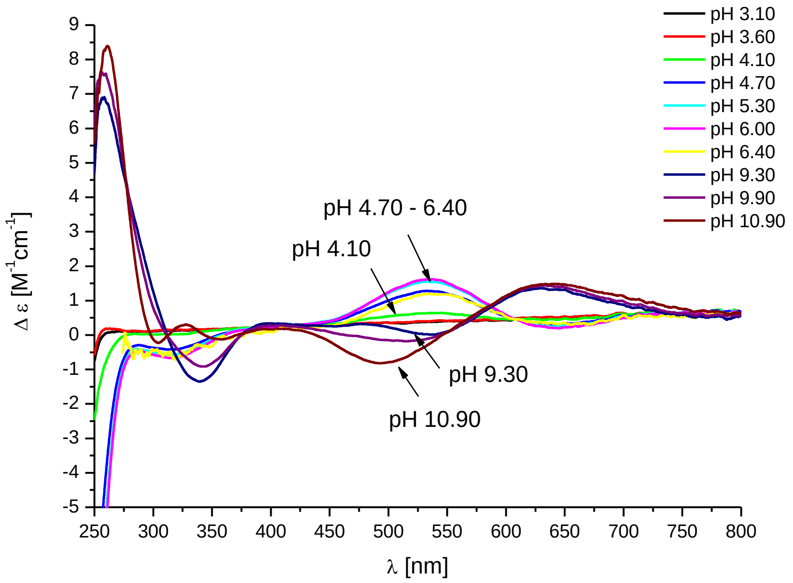

Figure S 9. CD spectra for Cu^{2+}-MB6complex in 30% DMSO solution. [Cu^{2+}] = 0.83 mM. Cu^{2+} to ligand ratio of 1:1.2. Cuvette path length 10 mm.

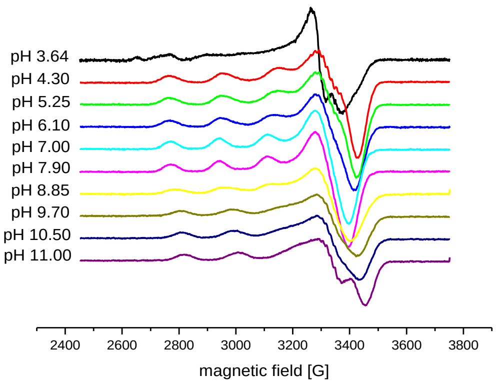

pH	$A_{\parallel}$ [G]	$g_{\parallel}$	$g_{\perp}$
3.00	120.6	2.42	2.08
3.64	-	-	-
4.30	186.3	2.24	2.05
5.25	186.3	2.24	2.05
6.10	184.2	2.24	2.05
7.00	171	2.26	2.045
7.90	171	2.26	2.045
8.85	181	2.24	2.045
9.70	183	2.22	2.045
10.5	184	2.22	2.045
11.0	190.8	2.205	2.04

Figure S 10. EPR spectra of Cu^{2+}- MB6 system in 30% DMSO solution of ethylene glycol (30%) at different pH values, T = 77 K (X-band – 9.5 GHz). [Cu^{2+}] = 1 mM; molar ratio M:L – 1:1.2.

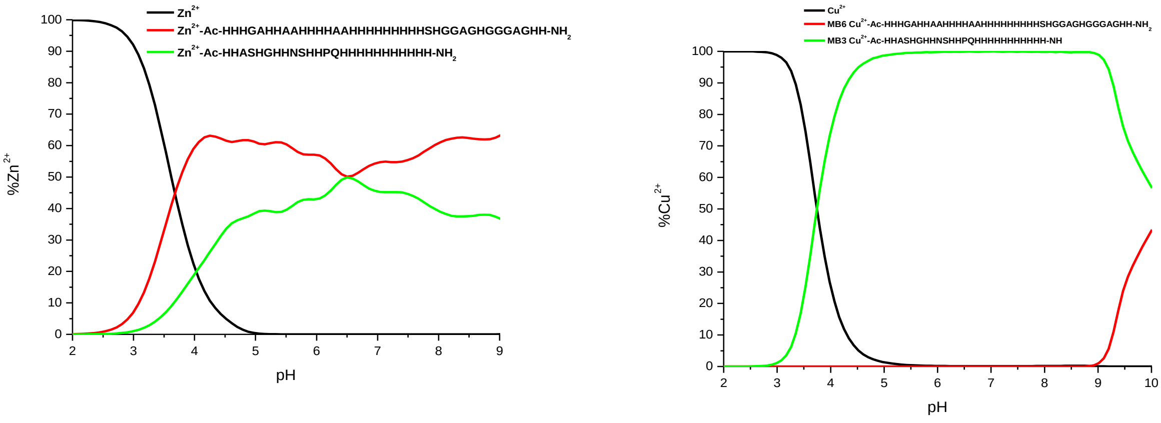

Figure S 11. Competition plots for (A) Zn^{2+}-Ac-HHASHGHHNSHHPQHHHHHHHHHHH-NH_2 (MB3) and Zn^{2+}-Ac-HHHGAHHAAHHHHAAHHHHHHHHHHSHGGAGHGGGAGHH-NH_2 (MB6); (B) Cu^{2+}-Ac-HHASHGHHNSHHPQHHHHHHHHHHH-NH_2 (MB3) and Cu^{2+}-Ac-HHHGAHHAAHHHHAAHHHHHHHHHHSHGGAGHGGGAGHH-NH_2 (MB6) complexes. Previously calculated stability constants are applied to a theoretical situation, in which equimolar amounts of Cu^{2+}/Zn^{2+} and all ligands are present.

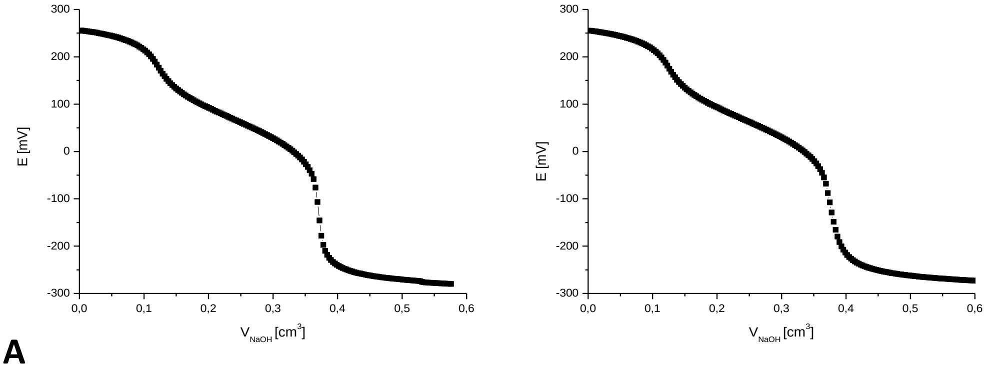

A

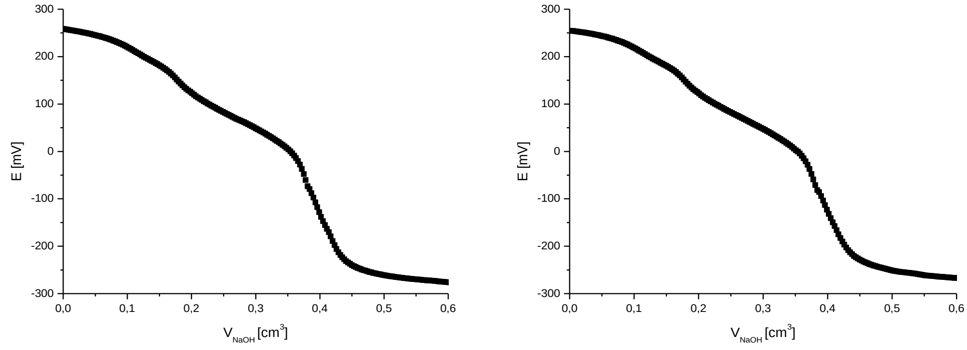
E [mV]
V NaOH [cm3]
E [mV]
V NaOH [cm3]

B

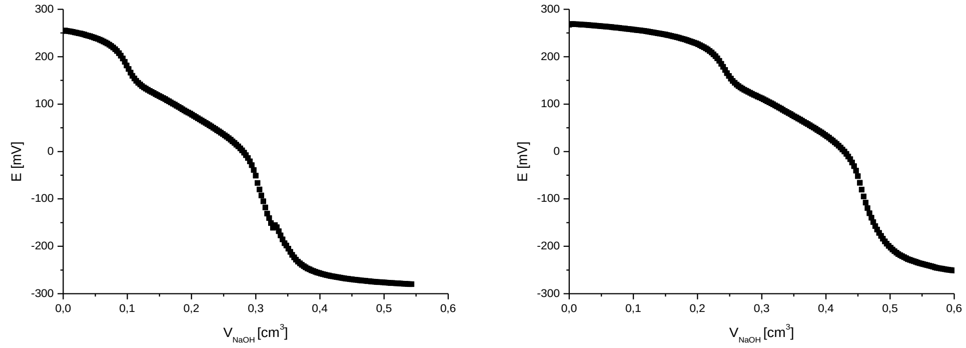
E [mV]
V NaOH [cm3]
E [mV]
V NaOH [cm3]

C

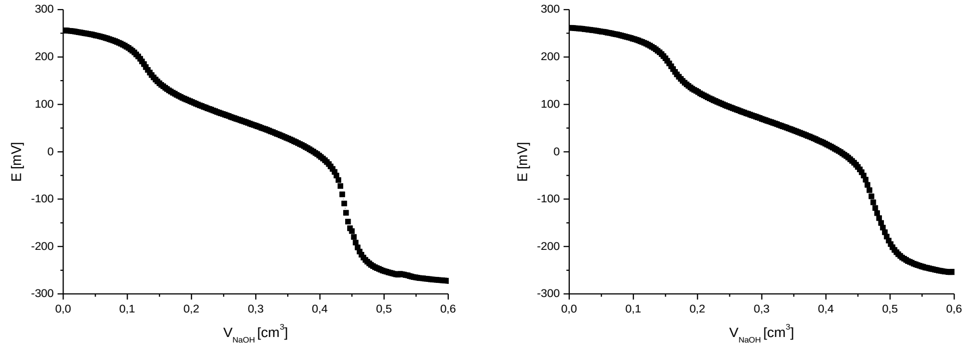
E [mV]
V NaOH [cm3]
E [mV]
V NaOH [cm3]

D

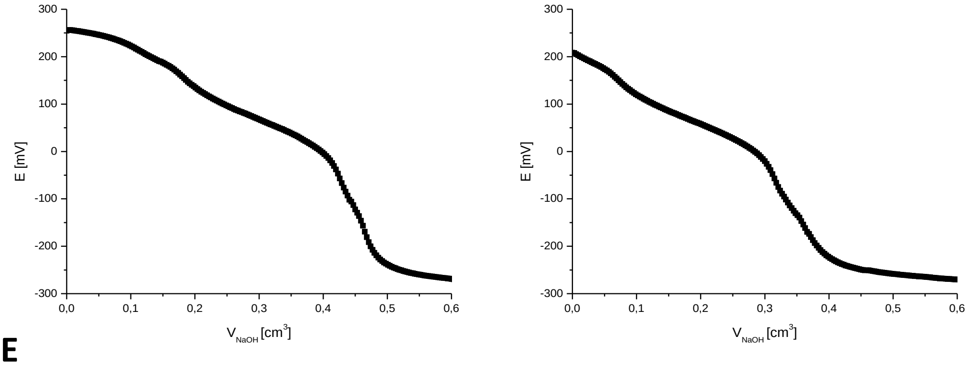
E [mV]
V NaOH [cm3]
E [mV]
V NaOH [cm3]

E

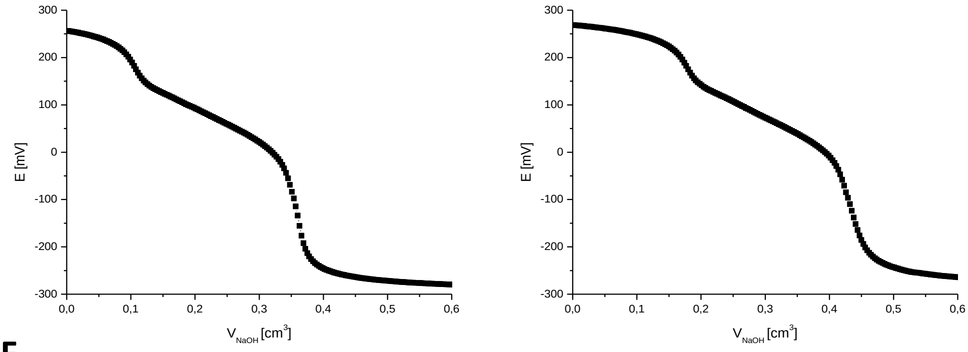

F

Figure S 12. Titration curves of the MB3 (A), Cu^{2+}-MB3 (B), Zn^{2+}-MB3 (C), MB6 (D), MB6-Cu^{2+} (E) and MB-Zn^{2+} (F) systems. [L] = 0.5 mM; molar ratio M:L – 1:1.2 – for Cu^{2+} complexes and 1:2 for Zn^{2+} complexes.
